# In-silico Assessment of Protein-Protein Electron Transfer. A Case Study: Cytochrome c Peroxidase – Cytochrome c

**DOI:** 10.1371/journal.pcbi.1002990

**Published:** 2013-03-21

**Authors:** Frank H. Wallrapp, Alexander A. Voityuk, Victor Guallar

**Affiliations:** 1 Department of Life Sciences, Barcelona Supercomputing Center, Nexus II Building, Barcelona, Spain; 2 Department of Computational Chemistry, University of Girona, Girona, Spain; 3 Institució Catalana de Recerca i Estudis Avançats, Barcelona, Spain; University of Wisconsin, Madison, United States of America

## Abstract

The fast development of software and hardware is notably helping in closing the gap between macroscopic and microscopic data. Using a novel theoretical strategy combining molecular dynamics simulations, conformational clustering, *ab-initio* quantum mechanics and electronic coupling calculations, we show how computational methodologies are mature enough to provide accurate atomistic details into the mechanism of electron transfer (ET) processes in complex protein systems, known to be a significant challenge. We performed a quantitative study of the ET between Cytochrome c Peroxidase and its redox partner Cytochrome c. Our results confirm the ET mechanism as hole transfer (HT) through residues Ala194, Ala193, Gly192 and Trp191 of CcP. Furthermore, our findings indicate the fine evolution of the enzyme to approach an elevated turnover rate of 5.47×10^6^ s^−1^ for the ET between Cytc and CcP through establishment of a localized bridge state in Trp191.

## Author Summary

We have developed a protocol capable of describing long-range electron transfer mechanisms at an atomic detailed level. We demonstrate the maturity of the computational techniques in obtaining a quantitative view of the Cytochrome c Peroxidase/Cytochrome c electron transfer process, known to be a significant challenge. In excellent agreement with experimental data, our results allow for the description of the electron transfer pathway, its mechanism and the electron transfer rate at a quantitative level. The overall protocol is free of parameterization and can be applied to any complex electron transfer process. Furthermore, the results reveal the fine enzyme evolution of this protein-protein complex to optimize its electron transfer rate by a localized bridge state.

## Introduction

Electron transfer (ET) is a fundamental reaction in biochemistry [1], [2]. Its comprehensive elucidation is crucial for the understanding of biological function and the design of synthetic energy transduction systems. In this matter, the question of how intermediary medium controls the electron transfer process between two redox proteins remains an intriguing and challenging problem in biophysics and biochemistry [3]–[7]. In general, ET can be considered as a transition between two electronic states, donor (D) and acceptor (A). Following Marcus theory, the ET rate is determined by the electronic coupling between D and A (V_DA_), the reaction free energy (ΔG) and the reorganization energy (*λ*) needed to adopt the system from one state to the other:
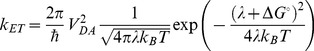
(1)Besides direct and bridge-mediated superexchange between donor and acceptor, ET can also occur through incoherent hopping including transiently populated electronic states localized on a bridge [8].

**Figure 1:**
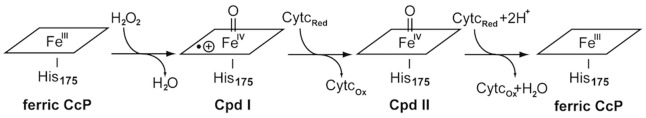
Catalytic cycle of CcP.

**Figure 2:**
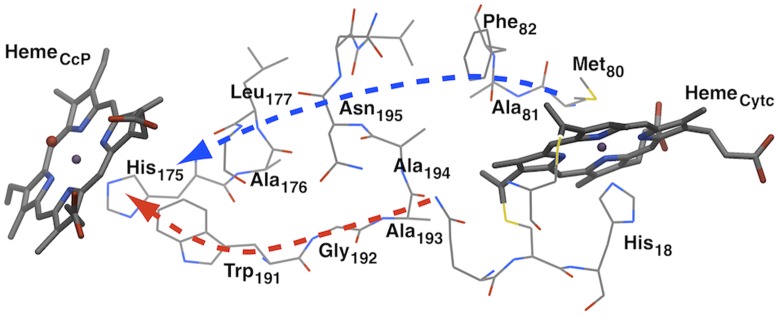
Electron transfer region of the CcP/Cytc complex. The ET pathway proposed by Pelletier and Kraut is shown in red, the ET pathway suggested by Siddarth is shown in blue.

There have been many investigations on ET in enzymes [9]–[13], with several of them on ruthenium-modified proteins [14]–[28]. In terms of the theoretical study of ET in protein, the *Pathways* method developed by Beratan et al. has made considerable impact in the 90's [1], [29], while it got replaced by the combined QM-MD approach [30]–[33], being employed in this study. Of special interest is the ET in the Cytochrome c Peroxidase/Cytochrome c (CcP/Cytc) complex [9], [34]–[39]. In its catalytic cycle, CcP undergoes a two-electron oxidation by peroxide forming an oxo-ferryl intermediate called compound I (CpdI), which is then reduced by two distinct Cytc to firstly CpdII and finally the ferric resting state again (see Figure 1). The measurement of the pure ET rate (not including the complex formation) still bears difficulties due to instrumental dead-times. It was estimated to be greater than 2000 s^−1^ measured by stopped flow experiments on the ground state ET in the covalently linked complex, or rather 5.0×10^4^ s^−1^ measured by flash photolysis methods using Ru-porphyrin photo-excited states of the noncovalent complex [38], [40]. Pelletier and Kraut proposed a σ-bond tunneling pathway from Cytc to Trp191 following the residues Ala194, Ala193 and Gly192 of CcP (shown in red in Figure 2), which is supported by several other studies [34], [36], [41], [42]. Furthermore, it is widely accepted that CpdI oxidizes Trp191 generating the radical intermediated Trp191^+^, which then is reduced by Cytc [43]–[48]. Consequently, the ET mechanism can be considered as hole transfer (HT).

Understanding the ET mechanism at an atomic detailed level is a crucial step towards rational enzyme engineering. In this paper we demonstrate how the evolution of computational methodologies allows today for a comprehensive study on complex protein-protein ET. Using a new theoretical strategy combining conformational sampling, a recently developed ET pathway search (QM/MM e-Pathway) [49], [50] and robust *ab-initio* quantum mechanics electronic coupling calculations, we perform a comprehensive case study of the ground state ET between CcP and its redox partner Cytc. In addition to the identification of the ET mechanism and pathway, this study provides a computational assessment of the ET rate constant. Our analysis confirms the underlying ET mechanism to be sequential hopping, with the Trp191 radical cation as an intermediate state. This finding provides a select example of enzyme evolution by means of breaking down slow processes into several faster ones, together with a fine-tuning of the ΔG and *λ* differences, thereby gaining total turnover rate.

## Results/Discussion

The overall simulation protocol proposed here involves: 1) the conformational sampling of the protein-protein complex, 2) the analysis of possible electron transfer pathways, and 3) the computation of V_DA_, ΔG and *λ* to obtain the ET rate for the different pathways found in step 2.

### Conformational sampling

Molecular dynamics techniques have experienced a significant advancement in the recent years. Both the development of better algorithms together with the availability of a larger number of processors make it easy to run tens of nanoseconds in few days on a small local cluster (16–32 processors). Furthermore, hundreds of nanoseconds or even microseconds are becoming a possibility with the usage of GPU clusters or specialized purpose machines [51], [52]. These long MD runs have also provided the necessary phase space exploration to further optimize the force fields [53].

For CcP/Cytc, the existence of a “rigid” engineered covalent cross-link protein-protein complex [38], with very similar activity and structure to the non-covalent (wild type) complex, points to the existence of a main ET conformation. Thus, a relatively short MD around the initial crystal should provide representative conformations. Nevertheless, we run a 30 ns MD trajectory that confirmed the stability of the protein-protein complex. Additionally the ET rate results (see below) confirm the properness of the conformational sampling.

Starting from the Pelletier and Kraut crystal structure [34] we performed 30 ns of molecular dynamics (MD) on the complex and extracted 10 snapshots within the first 2 ns (based on clustering of the ET region RMSD) for local sampling and 3 more snapshots at time steps 10 ns, 20 ns and 30 ns for nonlocal sampling. The RMSD for the heme groups as well as the superposition of all 14 conformations (crystal structure plus 13 snapshots from MD) is given in Figure S1 and S2 in Text S1. The overall results indicate clearly the stability of the complex and the lack of large fluctuations in the donor, acceptor and interphase region.

### ET pathway

**Figure 3:**
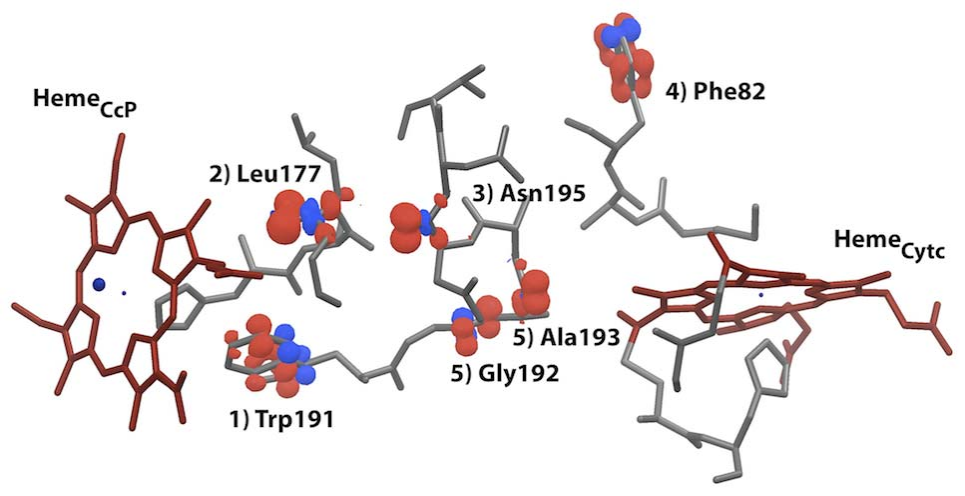
Visual output of the QM/MM e-Pathway method applied on a single MD snapshot. Molecular orbitals (spin densities of electron hole) are labeled by corresponding residue name and iteration of the approach. I.e. Trp191 being identified first, then excluded from QM region, such that Leu177 is identified second, etc. See methods and SI for details on the QM/MM e-Pathway approach.

Following the conformational sampling, we identified the potentially important residues for the ET in each of the 14 snapshots. By using mixed quantum mechanics and molecular mechanics (QM/MM) techniques, it is now possible to track the electron pathway for complex biological systems. To this purpose, we developed the QM/MM e-Pathway, an iterative procedure capable of tracking the residues (molecular orbitals) with highest electron affinity in the transfer region (see Figure 3 and methods section).

**Figure 4:**
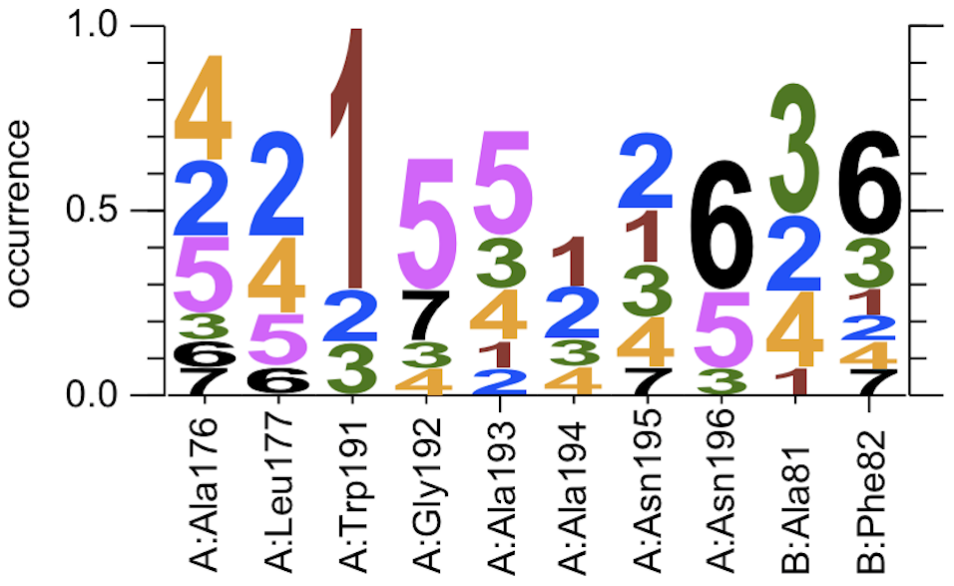
Logo plot summarizing the QM/MM e-Pathway calculations on all 14 snapshots, where the size of a digit *d* for residue *r* indicates the relative frequency of residue *r* being identified as hole acceptor at step *d* of the iterative approach.

In detail, the examined residues are Ala176, Leu177, Trp191, Gly192, Ala193, Ala194, Asn195 and Asn196 of CcP as well as Ala81 and Phe82 of Cytc, spanning the protein space between the donor and acceptor. The logo plot, given in Figure 4, summarizes the QM/MM e-Pathway calculations on all 14 snapshots, where the size of a digit *d* for residue *r* indicates the relative frequency of residue *r* being identified as hole acceptor at step *d* of the iterative approach. The total occurrence of a specific residue can deviate from 1.0 because multiple residues were sometimes identified within a single QM/MM e-Pathway step and we also stopped the search after 7 steps. The results clearly indicate that Trp191 is the first hole acceptor in 10 of the 14 conformations. Thus, from the QM/MM e-Pathway analysis, one can obtain qualitative mechanistic information. In this case, for example, it indicates the possibility of having Trp191 as the localized bridge state in a two-step, sequential hopping ET mechanism.

We denote a set of residues as the ET pathway for a specific snapshot, once a connecting chain of residues between the donor and acceptor is found through the stepwise application of the QM/MM e-Pathway approach (see supporting information). Our calculations result in 2 distinct ET pathways for the CcP/Cytc system. The first pathway *path1* consists of residues Trp191, Gly192, Ala193 and Ala194 of CcP, and is assigned to 10 of the 14 conformations. The second pathway *path2* consists of residues Ala176, Leu177, Ala194 and Asn195 of CcP, and Phe82 and Ala81 of Cytc, and is assigned to 4 of the 14 conformations. Comparison of our results with published data shows that *path1* is identical to the ET pathway proposed by Pelletier and Kraut and confirmed by several studies [1], [34], [42]. There is less evidence for *path2* to be involved in the ET between Cytc and CcP, yet it was proposed by Siddarth [54].

### V_DA_, ΔG, and *λ*

The QM/MM e-Pathway approach only identifies important residues in the ET region in a qualitative manner. Therefore, we computed the ET rate for each possible pathway and mechanism in order to get a quantitative measure. As mentioned above, based on the QM/MM e-Pathway analysis we also investigated the hole localization on Trp191 and its role in the ET process. Thus, the following text is split into two parts: 1) single-step HT between Cytc and CcP and 2) sequential hopping HT with Trp191 as localized bridge.

#### Single step HT

In order to investigate the single-step HT, we calculated the electronic coupling between donor and acceptor for all 14 conformations based on 4 different setups: 1) *direct* electronic coupling of heme_CcP_ and heme_Cytc_ only, 2) *full* bridge-mediated electronic coupling including the complete ET region, and bridge-mediated electronic coupling including residues of 3) *path1* and 4) *path2*. The different setups are applied by including the corresponding residues in the QM region of the mixed QM/MM calculations. The *rmsV_DA_* values are given in Table 1 while the single *V_DA_* values for each snapshot can be found in the supporting information.

**Table 1:** Average distances *d* in Å, Electronic coupling *rmsV* in eV, Δ*G*° in eV, λ in eV and in s^−1^ calculated for HT between donor and acceptor (DA), donor and bridge (DB), and bridge and acceptor (BA), respectively.

HT	Step	*d*	*rmsV* (*direct*)	*rmsV* (*full*)	*rmsV* (*path1*)	*rmsV* (*path2*)	Δ*G*°	*λ*	*k_ET_*
1-step	DA	27.2	5.81×10^−9^ (0.68)	3.04×10^−6^ (0.85)	3.71×10^−6^ (0.28)	3.99×10^−7^ (0.45)	−0.92	1.38 (0.85)	8.99×10^4^
2-step	DB	21.2	1.35×10^−7^ (0.82)	2.23×10^−5^ (0.66)	1.81×10^−5^ (0.72)		−0.55	0.88 (0.74)	5.47×10^6^
	BA	7.1	1.47×10^−2^ (0.81)				−0.37	0.19 (0.73)	1.78×10^12^

The electronic coupling is calculated applying QM setups *direct*, *full*, *path1* and *path2*. *k_ET_* is calculated by Marcus theory applying the respective highest electronic coupling of the system. Fluctuations are depicted through the coherence factor 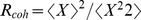 given in parentheses.

Clearly, the possible electron transfer between heme_CcP_ and heme_Cytc_ is of bridge-mediated nature. Due to the large distance of about 27 Å between donor and acceptor, the direct electronic coupling is very weak with *V_DA_*(*direct*)∼10^−9^ eV. In contrast, the bridge-mediated electronic coupling lies within the range of 10^−7^ to 10^−5^ eV, indicating that the intermediary protein scaffold enhances the ET transfer between donor and acceptor. In detail, the electronic coupling based on the *full* and *path1* transfer region are equally high, while the electronic coupling on *path2* is one order of magnitude lower, with *rmsV_DA_*(*path1*) = 3.04×10^−6^ eV, *rmsV_DA_*(*full*) = 3.71×10^−6^ eV and *rmsV_DA_*(*path1*) = 3.99×10^−7^ eV, respectively. The results also show that the electronic coupling values fluctuate only moderately due to thermal movements of the protein, indicating that the single-step HT is not conformational gated.

In order to obtain the ET rate from Cytc to CcP by Marcus theory, the energies Δ*G*° and *λ* have to be determined. From QM calculation of separate species CpdI, CpdII, Cytc_Ox_ and Cytc_Red_ in gas phase as well as implicit solvent, we obtained very similar values of Δ*E* = (*E*(CpdII)+*E*(Cytc_Ox_))−(*E*(CpdI)+*E*(Cytc_Red_)) of −0.93 and −0.95 eV, respectively. In addition, we performed energy minimization of the initial and final states of the whole protein complex by QM/MM methodology, resulting into Δ*E* = −0.92 eV. These energies are in excellent agreement with the experimental value of Δ*G*° = −0.9±0.15 eV found for the reaction of CcP_horse_ with Cytc [55]. This result indicates that the entropic effects play only a minor role in this process, why we substitute Δ*E* by Δ*G*° for the rest of this study (see Methods section). We estimated the reorganization energy *λ* = 1.38 eV through the vertical energy difference between the different electronic states by applying the method described by Blumberger [56]. This value is comparable to *λ* = 1.5 eV experimentally determined by Conklin et al. on the CcP/Cytc system [55], and also lies within the range of *λ* = 1.18–1.80 eV computationally predicted for the intra-protein ET system Ru(bpy)_2_(im)His33 cyt c [56] and *λ* = 1.2 eV measured on Ru((NH_3_)_4_)LHis33-Zn-cyt c [57]. Using Marcus theory on the ET parameters obtained, the rate constant of the single-step ET between CcP and Cytc is calculated to be 8.99×10^4^ s^−1^.

#### Two-step HT

The QM/MM e-pathway analysis indicated the importance of Trp191. Furthermore, we identified redox states localized on Trp191 within the energy window of the heme orbitals during the electronic coupling calculation of the single-step HT. It is also experimentally proven that Trp191 is able to localize a transient hole, forming a positive radical [47], [48], [58]. Thus, we split the ET process into two separate steps: donor-bridge (DB, Cytc to Trp191) and bridge-acceptor (BA, Trp191 to CcP) and calculated the electronic coupling for each of them.

The main step of the two-step process is HT from heme_Cytc_ to Trp191. Its electronic coupling through residues of *path1* is essentially the same as the *full* and significantly higher than the *direct* electronic coupling, thus indicating a bridge-mediated HT along *path1*, with *rmsV_DB_*(*full*) = 2.23×10^−5^ eV. The electronic coupling of the other step, Trp191 to heme_CcP_, is very strong with *rmsV_BA_*(*direct*) = 1.47×10^−2^ eV, as Trp191 is in direct vicinity to heme_CcP_. The rate of the total reaction is limited by the slowest step. Using QM/MM calculations as explained above, we estimated ΔG° = −0.55 eV and *λ* = 0.88 eV for the HT between Cytc and Trp191, resulting in *k_ET_* = 5.47×10^6^ s^−1^. Reduction of heme_CcP_ by Trp191 is very fast with ΔG° = −0.37 eV and *λ* = 0.19 eV, resulting in *k_ET_* = 1.78×10^12^ s^−1^. Consequently, the rate-limiting step is HT between Cytc and Trp191 of CcP.

### ET mechanism

Our results are in agreement with experiments in both the ET mechanism as well as the ET rate constant. From the literature it is known that CpdI oxidizes Trp191 generating the radical intermediated Trp191^+^, which then is reduced by Cytc [43]–[48]. Comparing our computed ET rates for the single- and two-step HT processes, it is clear that the electron hole is transferred between Cytc and CcP by sequential hopping with the intermediate state being Trp191^+^. Interestingly, when comparing the couplings and rates for the two-step and one-step processes, we observe a ∼60× larger rate constant for the two-step mechanism despite of only ∼6× larger electronic coupling. The reason for this is the relation between ΔG°, *λ* and *k_ET_* depicted in the Marcus equation, with *k_ET_* being maximal when *λ* = −ΔG°. The direct HT from Cytc to CcP has *λ* - (−ΔG°)  =  0.46 eV, whereas the rate-limiting HT between Trp191 and Cytc of the two-step process has *λ* - (−ΔG°) = 0.33 eV only, thus enabling higher rate constant at similar electronic coupling. These findings indicate the fine evolution of the enzyme to approach an increased turnover rate for the ET between CcP and Cytc through establishment of the localizable bridge state Trp191^+^.

We computed the rate of the ground state ET transfer between Cytc and CcP to be 5.47×10^6^ s^−1^. This result is in agreement with experimental ET rates to be greater than 2000 s^−1^, measured using stopped-flow methods on the ground state ET [38]. Although our results furthermore agree with ET rates measured to be greater than 5.0×10^6^ s^−1^ by flash photolysis on photo-exited ruthenium-modified Cytc [40], these values should not be compared directly due to the different kind of ET states involved.

As indicated by the coherent numbers in Table 1 (or by the individual values for the different snapshots in the supporting information), the coupling and reorganization energies for the different snapshots are quite consistent. Taking into account the computed rate constant, in good agreement with the experimental values, the low fluctuations in V_DA_ and *λ*, together with the small conformational changes along the MD, indicates a nongated ET mechanism. In terms of the reorganization energy, we are aware of the simplicity of our model and the importance of including protein electronic polarization into the calculations. Nevertheless, custom fit MD templates for both states RO and OR based on QM/MM optimization of the hemes plus ligated residues, as well as the rigidity of the interface and the fact that donor and acceptor are completely buried into protein and not accessible to solvent, lets us safely estimate correct reorganization energies as well as Δ*G*° through Δ*E*, as done in this study. However, the application of stated methodology to other ET systems might make specific adaptions necessary, such as more sampling or polarizable force fields to estimate *λ* and Δ*G*°.

In summary, we have performed a comprehensive mechanistic case study on the ET process in the CcP/Cytc complex. It shows that the maturity of computational techniques and a novel protocol combining conformational sampling, ET pathway mapping and calculation of the key ET parameters (ΔG°, V and *λ*), allows a detailed description of the underlying ET mechanism including the estimation of absolute rates of all relevant ET steps for this well-known system. Currently, there are efforts under way testing the approach in further systems in order to validate its general applicability. For the CcP/Cytc complex, our approach has confirmed a two-step nongated mechanism with residues Ala194, Ala193, Gly192 and Trp191 of CcP acting as the main ET pathway, with Trp191 serving as the transient hole acceptor at the first stage of the ET process. Most importantly, in the rate-limiting step of the two-step HT process, *λ* is found to be better matching -Δ*G*° than in the single-step HT, resulting in a much higher rate despite similar electronic coupling. This finding provides a select example of enzyme evolution by means of breaking down slow processes into several faster ones, thereby gaining total turnover rate.

## Materials and Methods

All QM/MM calculations were performed with Schrodinger's QSite program [59]. The spin-unrestricted DFT method with the M06 functional [60] was applied for the QM/MM geometry optimization as well as for the analysis of ET mechanisms by using the QM/MM e-Pathway approach (see supporting information and references) [49], [50]. All setups included the 6–31G* basis set for main-group elements and the lacvp pseudo-potential for Fe. The DFT methodology does not include the dispersion corrections, which could slightly affect the geometries, However these corrections do not change the electron interaction in the system and therefore have no direct impact on the electronic coupling.

For our calculations, we chose the structure of the native CcP/Cytc complex derived by Pelletier and Kraut [34]. Protocols of the protein preparation, MD simulation and QM/MM energy minimization are given in the supporting information. In brief, the data of QM/MM calculations on both hemes in their reduced states were used to construct the diabatic states and derive the electronic coupling (see below). The resulting conformation is referred to as the crystal complex of CpdII/Cytc_RED_ since the proteins did not undergo conformational changes throughout the equilibration process. We performed 30 ns of molecular dynamics (MD) on the complex, applying GROMACS [61] together with the OPLS force field. Snapshots were extracted every 1 ps within the first 2 ns and furthermore at time steps 10 ns, 20 ns and 30 ns. Based on the RMSD of the ET region, we clustered all 2000 snapshots from the 2 ns trajectory by using the k-medoids algorithm [62] and chose the 10 resulting clustering modes as representatives. In particular, the selected conformations were taken at time steps 0, 162, 432, 990, 1083, 1356, 1527, 1764, 1814 and 1884. Together with the crystal complex and snapshots at 10, 20 and 30 ns, our study included 14 conformations.

ET pathways are identified through the recently developed QM/MM e-Pathway approach [49], [50], where the ET region between the donor and acceptor is described by QM and the remainder of the protein by MM level of theory. For details we refer to the supporting information. In brief, the iterative procedure starts by finding the first acceptor of the hole through localization of the spin density in the ET region, given by a single point calculation of the system having one electron missing and thus a doublet spin state. In the next iteration, the previously identified residue is excluded from the QM region, turning it into a classical residue. Therefore, an electronic description of this residue is no longer possible and the electron hole needs to find its next host. A set of residues is designated as an ET pathway, once a connecting chain of residues between the donor and acceptor is found through the stepwise application of the approach.

The electronic coupling values were calculated by the FCD method [24], [63]–[65] in combination with Koopmans' theorem [66]. Here, the properties of the adiabatic states get approximated through one-electron energies and the highest occupied molecular orbitals localized on the donor and acceptor sites in the corresponding neutral system. It is known, that DFT calculations of open-shell systems leads to artificial delocalization of the unpaired electron because of incomplete cancellation of the electron self-interaction [67], [68]. It means that excess charge delocalization in radical cations and radical anions computed by DFT may be considerably overestimated. It was shown, however, for different functionals that the excess charge distribution is well described by Kohn-Sham orbitals of neutral dimers [69]. An alternative promising approach is the construction of the diabatic Hamiltonian using charge-localized broken-symmetry states [70]. The performance of this scheme is still not well established. In particular, it was found that calculated values of electronic couplings may be extremely sensitive to the choice of the functional [71]. Another important point is the performance of the *two-state* model based on a unitary transformation of adiabatic states to diabatic states. A general consideration has been revealed that the coupling derived with the two-state FCD scheme accounts properly for both the direct and bridge mediated superexchange interaction of the donor and acceptor in donor-bridge-acceptor systems [72].

All *rmsV* values were computed following reference [73], applying 2 (nearly) degenerate states for the donor (N*_D_*) and acceptor (N*_A_*) site, respectively, and 1 for the bridge site. We note that ET from any initial state of the donor can occur to *any* of the N*_A_* final states of the acceptor (N*_A_* parallel ET processes); thus the rate computed with *rmsV* must be multiplied by N*_A_*. Alternatively, the effective electronic coupling can be defined as 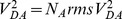. We refer to direct coupling when the QM region consists solely of the donor and acceptor sites, and to bridge mediated coupling when the QM region also includes several specified bridging residues.

The ET rate was calculated using Marcus expression [74], where we estimated the driving force *ΔG*° as the energy change Δ*E* = *E*_products_ - *E*_reactants_. QM geometry optimization of separated species CpdI, CpdII, Cytc_Ox_ and Cytc_Red_ was performed in the gas phase as well as in the implicit solvent with the dielectric constant of 4.0 (to simulate the protein environment). In addition, we also computed the optimal geometry of the CpdI - Cytc_Red_ (reactant) and CpdII - Cytc_Ox_ (product) state using the QM/MM approach. The neglected entropy contribution of ΔG° appears to be small; we did not find significant conformational changes accompanying the ET reaction. In order to estimate the reorganization energy *λ*, we ran 4 ns of MD on both electronic states RO and OR of each donor-acceptor pair DA, DB and BA, respectively. We calculated the vertical energy difference Δ*E_ET_ = E_RO_ – E_OR_* for 5 randomly chosen snapshots from the MD simulation and estimated *λ* using 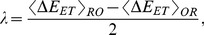
(2)where the brackets indicate averages over snapshots of MD either for the RO or the OR states, as specified by the subscripts [56].

## Supporting Information

Text S1 Supporting information on preparation of the CcP/Cytc complex, MD simulation on the CcP/Cytc complex, QM/MM e-Pathway and ET parameters.
(DOCX)
