# Supplementary material for: In-silico Assessment of Protein-Protein Electron Transfer. A Case Study: Cytochrome c Peroxidase – Cytochrome c
Source: PLoS Comput Biol. 2013 Mar 21;9(3):e1002990. doi: 10.1371/journal.pcbi.1002990 (PMC3605091; doi:10.1371/journal.pcbi.1002990)
Supplement: Text S1 — Supporting information on preparation of the CcP/Cytc complex, MD simulation on the CcP/Cytc complex, QM/MM e-Pathway and ET parameters. (DOCX) [file pcbi.1002990.s001.docx]

Supplementary Text S1

**In-silico Assessment of Protein-Protein Electron Transfer.**

**A Case Study: Cytochrome c Peroxidase – Cytochrome c**

Frank H. Wallrapp, Alexander A. Voityuk, and Victor Guallar

**Table of content:**

Preparation of the CcP/Cytc complex 1

MD simulation on the CcP/Cytc complex 1

QM/MM e-Pathway 3

ET parameters 3

References 6

## Preparation of the CcP/Cytc complex

We took chain A (Ccp) and B (Cytc) from the Protein Data Bank structure 2PCC for our calculations. We manually replaced the existing sulfate ligand of CcP by the oxo ligand being 6-coordinated to the Fe(IV) of CpdII. The protonation states of the histidines in CcP and Cytc were assessed by visual inspection. In particular, histidines 6, 52 and 60 of CcP and 39 of Cytc were protonated on the delta position, histidines 96 of CcP and 26 of Cytc were protonated on the epsilon position and histidines 181 of CcP and 33 of Cytc were doubly protonated. The system was soaked and equilibrated using Schrodinger’s Impact ([1](#_ENREF_1)), applying OPLS and the SPC solvent model together with 100 ps of molecular dynamics (MD) following a truncated Newton minimization. Both hemes and their ligated residues were kept frozen during the equilibration process. A layer of 10 Å of water around the proteins was kept as solvent. In order to gain the exact conformation of the system having both active sites in the reduced oxidation state, we QM/MM minimized the system in an oscillating approach. First we QM/MM minimized CcpII by having the heme, the oxo atom and the ligated His175 in the QM region while keeping frozen Cytc as well as all waters more than 5 Å away from CcP. For this, we used Schrodinger’s QSite ([2](#_ENREF_2)) applying DFT/B3LYP with the 6-31g*/lacvp basis set. The QM/MM boundary was treated by a hydrogen cut at the sidechain of His175. Within a second step we QM/MM minimized Cytc in the reduced state by only having the heme and its ligated residues in the QM region (-2 charge, singlet spin state) while keeping frozen CcP as well as all waters more than 5 Å away from Cytc. The QM/MM boundary was defined by a hydrogen cut at the sidechains of His18 and Met80 of Cytc as well as at the cysteine bridges of Cys14 and Cys17 being linked to the heme_Cytc_. The procedure was repeated between CcP and Cytc until final convergence of both. We applied the same approach on the CcP/Cytc complex for the different oxidation states applied in this study.

Table S1: QM setups for each system. Values given are formal charge and 2spin for each component as specified in QSite for each system.

| System | CcP | | | Trp | Cytc | | Total |
| --- | --- | --- | --- | --- | --- | --- | --- |
|  | Fe | Oxo | Porphyrin |  | Fe | porphyrin |  |
| CpdII-Trp_RED_/Cytc_RED_ | +4/+1 | -2/+1 | -4/0 | 0/0 | +2/0 | -4/0 | -4/+2 |
| CpdI-Trp_RED_/Cytc_RED_ | +4/+1 | -2/+1 | -3/-1 | 0/0 | +2/0 | -4/0 | -3/+1 |
| CpdII-Trp_OX_/Cytc_RED_ | +4/+1 | -2/+1 | -4/0 | +1/-1 | +2/0 | -4/0 | -3/+1 |
| CpdII-Trp_RED_/Cytc_OX_ | +4/+1 | -2/+1 | -4/0 | 0/0 | +3/+1 | -4/0 | -3/+3 |

## MD simulation on the CcP/Cytc complex

In order to apply the molecular dynamics software GROMACS together with the OPLS force field we created residue templates for CpdI, CpdII, Cytc_OX_, Cytc_RED_ and Trp_OX_. We took the ESP charges, atom lengths and angles derived from the QM/MM minimized structures. Force constants were taken from an OPLS heme-dioxy template published by Gogonea *et al.* ([3](#_ENREF_3)). The templates were modified to incorporate the oxo atom and ligated His175 for CcP and ligated His18 and Met80 as well as the cystein bridges of Cys14 and Cys17 for Cytc. Force constants involving the six-coordinated irons were taken from the literature ([4](#_ENREF_4)) respectively defined through vibrational analysis on a QM model of CpdII in the case of the iron-oxo bond (v=954.9, mass=12.4, k=666105.5 kJ/(mol nm2)). Starting from the QM/MM minimized structure of CpdII/Cytc_RED_, we performed a 30 ns NVT trajectory of the system in aqueous solvent at 289.15 K, following solvation in SPC including a 0.15 M salt concentration, a truncated Newton minimization and 20 ps of equilibration. Snapshots were saved every single ps for the first 2 ns as well as every 10 ns. We plotted the RMSD of the porphyrin and the 6-coordinated atoms of heme_CcP_ (panel A in Figure S1) and heme_Cytc_ (panel B in Figures S1) against their respective QM/MM templates throughout the first 2 ns of the MD simulation. The RMSD of heme_CcP_ has a mean value of 0.12 Å with a standard deviation of only 0.03, indicating very little fluctuations of the heme throughout the MD simulation. For heme_Cytc_ we have a similar mean RMSD of 0.13 Å and a standard deviation of 0.02, showing even less fluctuation in the trajectory. From both results we take that heme_CcP_ as well as heme_Cytc_ stay in their native conformation and no QM/MM minimization is needed for the electronic coupling calculations. Additionally we provide the superposition of all 14 conformations of CpdII/Cytc_RED_ used throughout this study in Figure S2.

For the reorganization energy calculation we also performed short MD simulations on the system in three different oxidation states. In detail we run 4 ns of MD on CpdI-Trp_RED_/Cyt_RED_, CpdII-Trp_OX_/Cytc_RED_ and CpdII-Trp_RED_/Cytc_OX_.


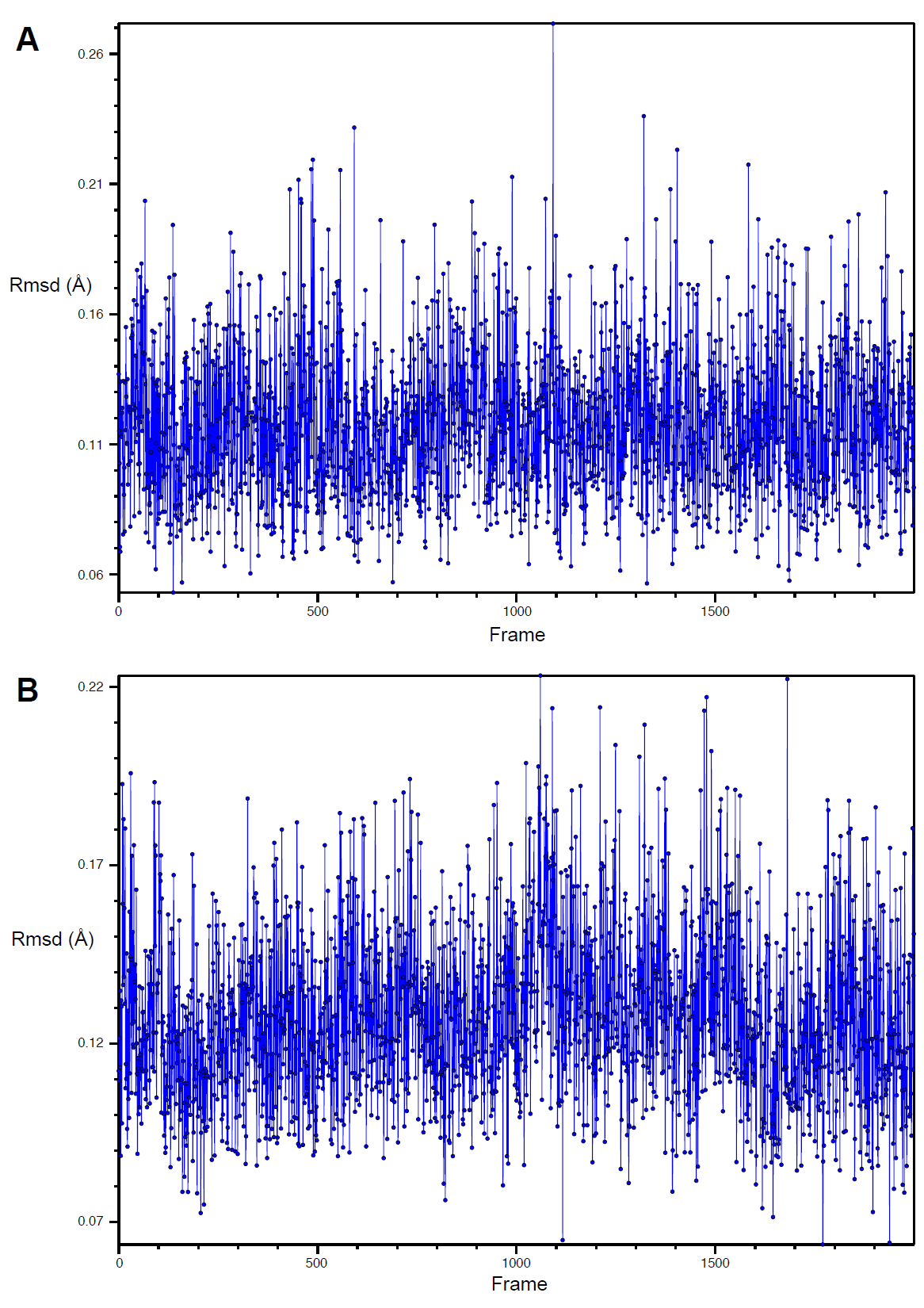


Figure S1: RMSD of hemeCcP (panel A) and hemeCytc (panel B) against their QM/MM respective templates throughout the MD simulation.


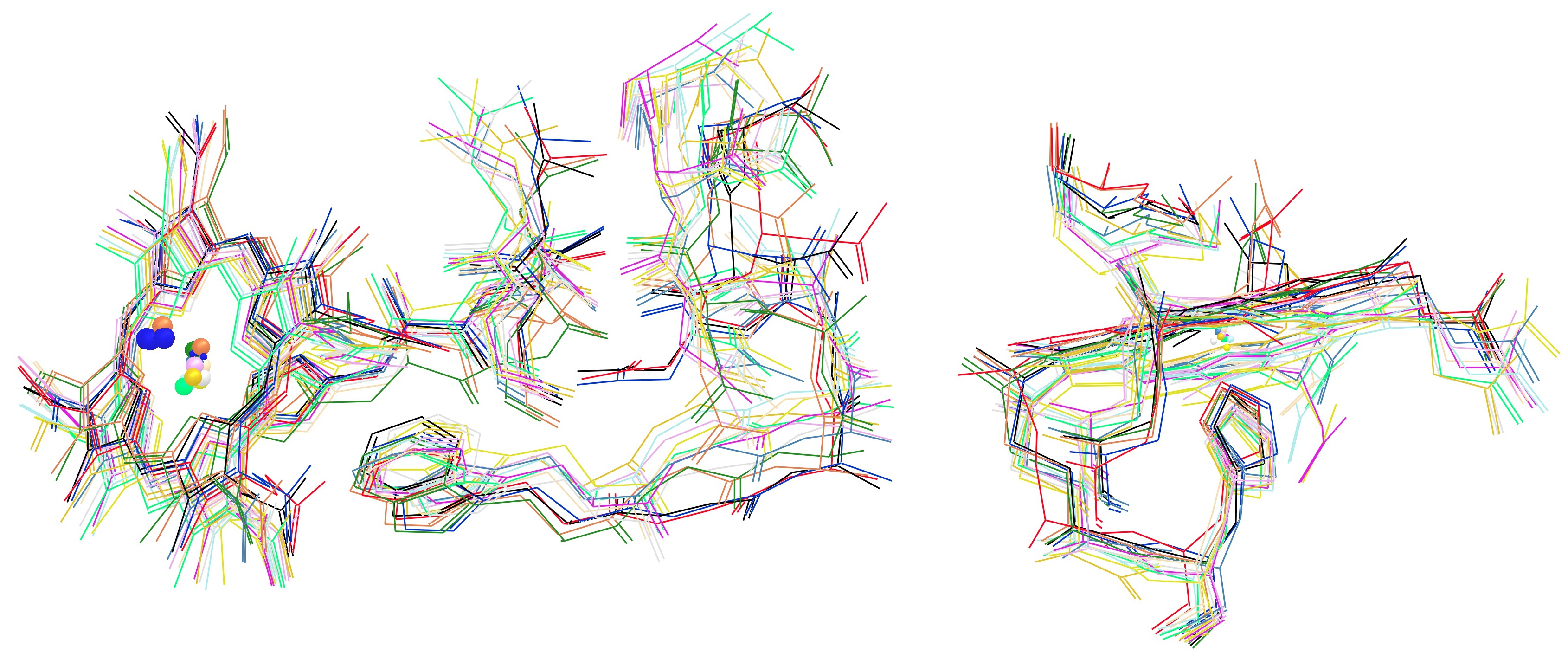


Figure S2: Superposition of all 14 conformations used for the QM/MM e-Pathway and electronic coupling calculations.

## QM/MM e-Pathway

In order to track electron migration, we recently proposed a new algorithm called the QM/MM e-Pathway ([5](#_ENREF_5), [6](#_ENREF_6)). The underlying idea is that regions contained in the MM part cannot allocate electrons due to the lack of electronic description. Hence, if an unpaired electron is present in the system it must necessarily occupy the quantum region. The method’s strategy is based on modifying the QM region following the evolution of the spin density. In case of an electron transfer pathway between two distant redox centers the methodology consists of the following procedure. As the initial setup, the parameters for the donor and the acceptor in the neutral state are derived, meaning both sites are reduced in our case of hole transfer. Here, the acceptor has taken itself an electron from the ET region but the donor has not given his electron away, yet. The parameterization consists of a QM/MM energy minimization of the donor and acceptor (both in their respective reduced state) from which we extract the geometry and atomic charges from the electrostatic potential. This parameterization is important because in the next step of the procedure both residues are going to be included only in the classical region, now only influencing the QM region by structural constraints as well as electrostatic and van der Waals interactions. Thus, there is going to be no electronic description of the donor and acceptor. Instead, the focus is on the ET region in-between, the region that now contains the electron hole. After this setup an iterative process is started where initially the entire transfer region is included in the QM region of the QM/MM calculation. The first acceptor of the hole is found by the localization of the spin density given by a single point calculation of the system missing one electron thus having a doublet spin state. Proceeding to the next iteration, the previously identified residue is turned into a classical residue by excluding it from the quantum region. In doing so, the method does not allow for an electronic description of it and thus, the hole needs to find its next host. The QM/MM boundary is treated by hydrogen cuts placed onto the backbone of the protein residues. The procedure is repeated until the identified residues connect to a direct pathway between the donor and acceptor.

## ET parameters

**Electronic coupling.** We computed the electronic coupling *V* using the Fragment charge method (FCD) in combination with Koopmans’ theorem, for which we refer to the original literature ([7-9](#_ENREF_7)). The RMSD of the donor and acceptor sites from the QM/MM optimized structures along the MD simulation revealed very little fluctuation, why we applied FCD on snapshots directly derived from MD on CpdII-TrpRED/CytcRED without further QM/MM optimization. In addition, we tested a complete QM/MM minimization of heme_CcP_ and heme_Cytc_ in their respective reduced states of snapshot 0 and computed the electronic coupling on the resulting conformation. Our calculations show that the electronic coupling does not change much from before to after the minimization and always lies within the range of the *rmsV_DA_* of all 14 conformations under study. Direct: *V_DA_*(noOpt) = 9.19×10^-9^ against *V_DA_*(QM/MM-Opt) = 6.38×10^-9^ with *rmsV_DA_*(14conf) = 5.81×10^-9^; path1: *V_DA_*(noOpt) = 1.30×10^-5^ against *V_DA_*(QM/MM-Opt) = 4.00×10^-6^ with *rmsV_DA_*(14conf) = 3.71×10^-6^; path2: *V_DA_*(noOpt) = 1.76×10^-7^ against *V_DA_*(QM/MM-Opt) = 2.56×10^-7^ with *rmsV_DA_*(14conf) = 3.99×10^-7^. Note that, unlike *∆Gº*, the electronic coupling is not significantly affected by external electric field but mainly depends on the character of the corresponding overlapping orbitals, as mentioned in the literature before ([8](#_ENREF_8), [10](#_ENREF_10)).

All *rmsV* values were computed following reference ([11](#_ENREF_11)), applying 2 (nearly) degenerate states for the donor = heme_Cytc_ (*N_D_*) and acceptor = heme_CcP_ (*N_A_*) site, respectively, and 1 for the bridge site = Trp191. Importantly, ET from any initial state of the donor can occur to *any* of the *N_A_* final states of the acceptor (*N_A_* parallel ET processes), thus the rate constant computed with *rmsV* must be multiplied by *N_A_*. Alternatively, the effective electronic coupling can be defined as $V_{DA}^{2}=N_{A}rmsV_{DA}^{2}$.

We refer to direct coupling when the QM region consists solely of the donor and acceptor sites, and to bridge mediated coupling when the QM region also includes several specified bridging residues. It is known that hydrogen cuts can disturb the partial charges of QM atoms in their vicinity ([12](#_ENREF_12)). Consequently, their exact placement is crucial for the correct application of FCD, being based on the charge separation of donor and acceptor sites. Preliminary tests have shown that placing the hydrogen cuts at least 3 bonds away from sites of interest do not affect their partial charges and result in correct coupling calculations. An example are hydrogen cuts from Trp191:N to Pro190:C and from Trp191:C to Gly192:N in order to incorporate the side chain of Trp191 into the QM region. The electronic coupling values of all systems for all conformations are given in Table S2 and Table S3.

**Gibbs free energy.** We estimated the driving force *∆Gº* as the energy change *∆E = E*_products_ - *E*_reactants_. We applied QM minimization of the separate ET transfer components CpdI, CpdII, Cytc_Ox_ and Cytc_Red_ in gas phase as well as in implicit solvent with a dielectric constant of 4.0 to simulate the protein environment. Additionally, we computed the optimal geometry of the CpdI - Cytc_Red_ (reactant) and CpdII - Cytc_Ox_ (product) state using the QM/MM approach. The neglected entropy contribution of ∆Gº appears to be very small because we did not find any significant conformational changes accompanying the ET reaction and our calculated values are in prefect agreement with experimental data given in the literature.

**Reorganization energy.** In order to estimate the reorganization energy λ, we applied the methods described by Blumberger ([13](#_ENREF_13)). We calculated the vertical energy difference ∆*E_ET_ = E_RO_ – E_OR_* for 5 randomly chosen snapshots from the MD simulation of all three ET processes, respectively: 1) Single-step HT between CcP and Cytc: CpdII/Cytc_OX_ vs. CpdI/Cytc_RED_; 2) First step between CcP and Trp191 of the two-step HT: CpdII-Trp_OX_ vs. CpdI-Trp_RED_; 3) Second step between Trp191 and Cytc of the two-step HT: Trp_RED_/Cytc_OX_ vs. Trp_OX_/Cytc_RED_. We then estimated λ using

,

where the brackets indicate averages over snapshots of MD either for the RO or the OR states of each system, as specified by the subscripts. We did very short QM relaxation of the redox centers of each snapshot (5 steps of QM/MM minimization with the MM part absolutely frozen) in order to eliminate internal strains that would generate unnaturally high energies. We want to point out that this approach basically only allows the wave function to relax and does not change the structure (RMSD of the conformations before and after relaxation is less than 0.01 Å). We furthermore carefully checked the spin state for each conformation after the relaxation in order to assure correct oxidation states of the species applied in the reorganization energies calculations. The precise parameterization of the redox centers in their respective oxidation state in combination with the unconstrained MD simulation of the complete protein complex, allows us to assume that the estimated λ derived by the above-mentioned approach includes both inner and outer sphere contribution. Detailed values of all RO and OR energies for the three different HT processes are given in Table S4.

Table S2: Electronic coupling values *rmsV_DA_* in eV calculated between donor and acceptor (DA) with QM setup *direct*, *full*, *path1* and *path2*, respectively. Donor-acceptor distance *d_DA_* in Å, measured between both irons.

| snapshot | *d_DA_* (Å) | DA, *direct* | DA, *full* | DA, *path1* | DA, *path2* |
| --- | --- | --- | --- | --- | --- |
| crystal | 26.4 | 5.48×10^-9^ | 2.41×10^-6^ | 1.35×10^-6^ | 1.17×10^-7^ |
| 0 | 26.7 | 9.19×10^-9^ | 3.03×10^-6^ | 1.30×10^-5^ | 1.76×10^-7^ |
| 162 | 27.3 | 4.42×10^-9^ | 3.94×10^-6^ | 8.94×10^-7^ | 9.99×10^-8^ |
| 432 | 27.3 | 1.26×10^-8^ | 3.72×10^-6^ | 6.28×10^-7^ | 2.85×10^-7^ |
| 990 | 26.9 | 4.28×10^-9^ | 3.40×10^-6^ | 2.36×10^-7^ | 3.65×10^-7^ |
| 1083 | 27.6 | 1.10×10^-9^ | 4.93×10^-6^ | 3.33×10^-6^ | 1.02×10^-7^ |
| 1356 | 27.2 | 3.10×10^-9^ | 1.72×10^-6^ | 5.17×10^-7^ | 3.65×10^-7^ |
| 1527 | 27.1 | 2.96×10^-9^ | 1.32×10^-6^ | 1.50×10^-6^ | 1.26×10^-6^ |
| 1764 | 27.7 | 5.74×10^-9^ | 1.06×10^-6^ | 8.89×10^-7^ | 4.90×10^-8^ |
| 1814 | 27.4 | 2.92×10^-9^ | 9.85×10^-7^ | 4.89×10^-7^ | 7.02×10^-8^ |
| 1884 | 27.3 | 4.05×10^-9^ | 3.35×10^-6^ | 1.03×10^-6^ | 3.56×10^-7^ |
| 10.000 | 27.2 | 2.99×10^-9^ | 3.47×10^-6^ | 5.69×10^-7^ | 2.18×10^-7^ |
| 20.000 | 27.0 | 4.08×10^-9^ | 3.17×10^-6^ | 1.29×10^-6^ | 1.15×10^-7^ |
| 30.000 | 26.7 | 9.58×10^-9^ | 9.58×10^-6^ | 1.81×10^-6^ | 1.68×10^-7^ |
| *rms* | 27.2 | 5.81×10^-9^ | 3.04×10^-6^ | 3.71×10^-6^ | 3.99×10^-7^ |
| *R_coh_* |  | 0.68 | 0.85 | 0.28 | 0.45 |

Table S3: Electronic coupling values *rmsV* in eV for HT between donor and bridge (DB), and bridge and acceptor (BA) with Trp191 as bridge. Distance *d* in Å, measured between center of aromatic side chain of Trp and iron of heme.

| snapshot | *d_DB_* (Å) | DB, *direct* | DB, *full* | DB, *path1* | *d_BA_* (Å) | BA, *direct* |
| --- | --- | --- | --- | --- | --- | --- |
| crystal | 21.4 | 1.97×10^-8^ | 1.16×10^-5^ | 2.98×10^-5^ | 6.8 | 2.06×10^-2^ |
| 0 | 21.2 | 8.66×10^-8^ | 1.01×10^-5^ | 9.86×10^-6^ | 7.1 | 1.30×10^-2^ |
| 162 | 21.3 | 6.08×10^-8^ | 2.03×10^-5^ | 5.11×10^-6^ | 7.1 | 1.04×10^-2^ |
| 432 | 21.2 | 1.20×10^-7^ | 8.34×10^-6^ | 9.77×10^-6^ | 7.2 | 1.20×10^-2^ |
| 990 | 20.6 | 1.43×10^-7^ | 1.93×10^-5^ | 1.52×10^-5^ | 7.1 | 1.00×10^-2^ |
| 1083 | 21.1 | 1.15×10^-7^ | 2.53×10^-5^ | 3.67×10^-5^ | 7.4 | 5.83×10^-3^ |
| 1356 | 21.2 | 2.00×10^-7^ | 9.35×10^-6^ | 3.62×10^-6^ | 7.0 | 7.55×10^-3^ |
| 1527 | 21.4 | 9.96×10^-8^ | 6.77×10^-6^ | 9.78×10^-6^ | 6.9 | 2.22×10^-2^ |
| 1764 | 21.6 | 2.02×10^-7^ | 1.04×10^-5^ | 1.02×10^-5^ | 7.1 | 1.44×10^-2^ |
| 1814 | 21.1 | 9.73×10^-8^ | 1.87×10^-5^ | 2.04×10^-5^ | 7.3 | 6.07×10^-3^ |
| 1884 | 20.9 | 6.68×10^-8^ | 1.12×10^-5^ | 1.20×10^-5^ | 7.0 | 1.14×10^-2^ |
| 10.000 | 21.6 | 1.79×10^-7^ | 2.65×10^-5^ | 6.33×10^-6^ | 7.0 | 1.48×10^-2^ |
| 20.000 | 21.4 | 2.21×10^-7^ | 5.74×10^-5^ | 2.79×10^-5^ | 7.3 | 6.93×10^-3^ |
| 30.000 | 21.4 | 1.00×10^-7^ | 6.31×10^-5^ | 1.79×10^-5^ | 7.0 | 2.89×10^-2^ |
| *rms* | 21.2 | 1.35×10^-7^ | 2.23×10^-5^ | 1.81×10^-5^ | 7.1 | 1.47×10^-2^ |
| *R_coh_* |  | 0.82 | 0.66 | 0.72 |  | 0.81 |

Table S4: Detailed values of all RO and OR energies for the three different HT processes: Single-step HT between CcP and Cytc directly, and the two separate steps of the two-step HT, HT between CcP and Trp191 and HT between Trp191 and Cytc. The species of each snapshot of the system is given in parenthesis (ie. RRO) with the fist letter indicating the oxidation state of CcP, the second of Trp191 and the third of Cytc. Coherence values are given in parenthesis for derived values.

|  | single-step HT | | two-step HT | | | | |
| --- | --- | --- | --- | --- | --- | --- | --- |
|  | *HT_CcP – Cytc_* | | | *HT_CcP-Trp191_* | | *HT_Trp191 - Cytc_* | |
|  | ∆*E_ET_* (OrR) | ∆*E_ET_* (RrO) | | ∆*E_ET_* (ORr) | ∆*E_ET_* (ROr) | ∆*E_ET_* (rOR) | ∆*E_ET_* (rRO) |
| snapshot 1 | 1.55 | -1.05 | | 0.11 | -0.01 | 0.32 | -1.22 |
| snapshot 2 | 1.32 | -1.74 | | 0.22 | -0.27 | 1.42 | -1.73 |
| snapshot 3 | 0.46 | -1.60 | | 0.20 | -0.60 | 1.01 | -0.28 |
| snapshot 4 | 2.30 | -1.97 | | 0.06 | -0.13 | 0.86 | -0.12 |
| snapshot 5 | 1.44 | -0.36 | | 0.15 | -0.20 | 1.30 | -0.51 |
| mean(∆*E_ET_*) | 1.41 (0.85) | -1.34 (0.84) | | 0.15 (0.60) | -0.24 (0.87) | 0.98 (0.86) | -0.77 (0.62) |
| Λ | 1.38 (0.85) | | 0.19 (0.73) | | | 0.88 (0.74) | |

## References

1. (2010) Impact (Schrödinger, LCC, New York, NY), 5.6.

2. (2010) QSite (Schrödinger, LCC, New York, NY), 5.6.

3. Gogonea V, Shy JM, & Biswas PK (2006) Electronic Structure, Ionization Potential, and Electron Affinity of the Enzyme Cofactor (6R)-5,6,7,8-Tetrahydrobiopterin in the Gas Phase, Solution, and Protein Environments. *J. Phys. Chem. B* 110(45):22861-22871.

4. Autenrieth F, Tajkhorshid E, Baudry J, & Luthey-Schulten Z (2004) Classical force field parameters for the heme prosthetic group of cytochrome c. *J. Comput. Chem.* 25(13):1613-1622.

5. Guallar V & Wallrapp F (2008) Mapping protein electron transfer pathways with QM/MM methods. *J. R. Soc. Interface* 5(suppl 3):233-239.

6. Wallrapp F, Masone D, & Guallar V (2008) Electron Transfer in the P450cam/PDX Complex. The QM/MM e-Pathway. *J. Phys. Chem. A* 112(50):12989-12994.

7. Rösch N & Voityuk AA (2004) Quantum Chemical Calculation of Donor–Acceptor Coupling for Charge Transfer in DNA. *Long-Range Charge Transfer in DNA II,* Topics in Current Chemistry), pp 37-72.

8. Voityuk AA & Rösch N (2002) Fragment charge difference method for estimating donor-acceptor electronic coupling: Application to DNA pi-stacks. *J. Chem. Phys.* 117(12):5607-5616.

9. Newton MD (1991) Quantum chemical probes of electron-transfer kinetics: the nature of donor-acceptor interactions. *Chem. Rev.* 91(5):767-792.

10. Wittekindt C, Schwarz M, Friedrich T, & Koslowski T (2009) Aromatic Amino Acids as Stepping Stones in Charge Transfer in Respiratory Complex I: An Unusual Mechanism Deduced from Atomistic Theory and Bioinformatics. *J. Am. Chem. Soc.* 131(23):8134-8140.

11. Voityuk AA (2010) Electron transfer between [4Fe-4S] clusters. *Chem. Phys. Lett.* 495(1-3):131-134.

12. Ferré N & Olivucci M (2003) The amide bond: pitfalls and drawbacks of the link atom scheme. *J. Mol. Struct. (Theochem)* 632(1-3):71-82.

13. Blumberger J (2008) Free energies for biological electron transfer from QM/MM calculation: method, application and critical assessment. (Translated from eng) *Physical chemistry chemical physics : PCCP* 10(37):5651-5667 (in eng).
